# Phylogenetic evidence for the ancient Himalayan wolf: towards a clarification of its taxonomic status based on genetic sampling from western Nepal

**DOI:** 10.1098/rsos.170186

**Published:** 2017-06-07

**Authors:** Geraldine Werhahn, Helen Senn, Jennifer Kaden, Jyoti Joshi, Susmita Bhattarai, Naresh Kusi, Claudio Sillero-Zubiri, David W. Macdonald

**Affiliations:** 1 Wildlife Conservation Research Unit, Department of Zoology, University of Oxford, The Recanati-Kaplan Centre, Tubney House, Tubney OX13 5QL, UK; 2 WildGenes Laboratory, Royal Zoological Society of Scotland, Edinburgh EH12 6TS, UK; 3 Centre for Molecular Dynamics Nepal CMDN, GPO Box 21049, Kathmandu, Nepal; 4 Resources Himalaya Foundation, Sanepa, Lalitpur, Nepal; 5 IUCN SSC Canid Specialist Group, Oxford, UK

**Keywords:** *Canis himalayensis*, *Canis lupus chanco*, Himalaya, Himalayan wolf, Nepal, phylogeny

## Abstract

Wolves in the Himalayan region form a monophyletic lineage distinct from the present-day Holarctic grey wolf *Canis lupus* spp. (Linnaeus 1758) found across Eurasia and North America. Here, we analyse phylogenetic relationships and the geographic distribution of mitochondrial DNA haplotypes of the contemporary Himalayan wolf (proposed in previous studies as *Canis himalayensis*) found in Central Asia. We combine genetic data from a living Himalayan wolf population collected in northwestern Nepal in this study with already published genetic data, and confirm the Himalayan wolf lineage based on mitochondrial genomic data (508 bp cytochrome *b* and 242 bp D-loop), and X- and Y-linked zinc-finger protein gene (ZFX and ZFY) sequences. We then compare the genetic profile of the Himalayan wolf lineage found in northwestern Nepal with canid reference sequences from around the globe with maximum likelihood and Bayesian phylogeny building methods to demonstrate that the Himalayan wolf forms a distinct monophyletic clade supported by posterior probabilities/bootstrap for D-loop of greater than 0.92/85 and cytochrome *b* greater than 0.99/93. The Himalayan wolf shows a unique Y-chromosome (ZFY) haplotype, and shares an X-chromosome haplotype (ZFX) with the newly postulated African wolf. Our results imply that the Himalayan wolf distribution range extends from the Himalayan range north across the Tibetan Plateau up to the Qinghai Lakes region in Qinghai Province in the People's Republic of China. Based on its phylogenetic distinction and its older age of divergence relative to the Holarctic grey wolf, the Himalayan wolf merits formal classification as a distinct taxon of special conservation concern.

## 1. Introduction

There are few studies on wolves in Central Asia and the taxonomic status of wolves in this region remains unresolved [1]. The available genetic evidence points towards the presence of two distinct wolf lineages in the region, the Mongolian grey wolf (*Canis lupus chanco,* Gray, 1863) and the Himalayan wolf (table 1) [6–8,11,12]. Aggarwal *et al*. [7] proposed ‘*Canis himalayensis*’ as scientific name for the Himalayan wolf, while Sharma *et al*. [6] refer to it as ‘*C. l. chanco*-Himalayan haplotype’. A study with 440 bp mitochondrial DNA (mtDNA) cytochrome *b* gene sequences using a molecular clock [6] estimated its time of divergence at more than 800 000 years before present. Recently, Chetri *et al*. [13] have found support for the presence of the Himalayan wolf lineage in Nepal with four scat samples collected in the Annapurna Conservation Area and analysed at the mtDNA control region. Nevertheless, the phylogeny, ecology and conservation status of the Himalayan wolf remains poorly understood [1], and proper recognition of it as a taxon is pending.

**Table 1.** Overview of discussed canid lineages with names, status and references. The grey wolf subspecies (*Canis lupus* spp.) were to date primarily described on the basis of geographic origin.

scientific name	common name	region	IUCN recognized	references
*C. lupus lupus*	Eurasian grey wolf	Europe, Asia	yes	[2,3]
*C. lupus chanco*	Mongolian grey wolf	Tibetan Plateau	no	[2,4,5]
*C. himalayensis*^a^ (*Canis lupus* *chanco*-Himalayan haplotype^b^)	Himalayan wolf	Himalayas, Tibetan Plateau	no	[6]^a^; [7]^b^
*C. lupus pallipes*	Indian grey wolf	Southwestern Asia, Middle east	yes	[3,5]
*Canis aureus lupaster/Canis lupus lupaster*	the currently proposed African (golden) wolf/formerly golden jackal	Northern Africa	no	[8–10]
*Canis aureus*	golden jackal	Eurasia	yes	[3]

The Holarctic grey wolf appeared in the Middle Pleistocene, approximately 800 000–300 000 years before present [14–16]. In the evolutionary history from the ancestors of the wolf-dog clade in the Early to Middle Pleistocene [15] to the contemporary Holarctic grey wolf, different wolf lineages such as the Himalayan wolf, the African wolf [8] and the Indian grey wolf *C. lupus pallipes* (Sykes, 1831) [6] diverged as monophyletic sister clades. The Holarctic grey wolf (*C. lupus* spp.), comprising different subspecies including the domestic dog *C. l. familiaris*, forms a relatively recent genetic lineage [2,9]. More basal is a distinct lineage which has been described as Himalayan wolf and the recently described African (golden) wolf (currently referred to as *Canis aureus lupaster*) [8,9,17]. Based on their phylogenetic reconstruction, Rueness *et al*. [10] propose that the Himalayan and African wolf lineages may have existed before the radiation of the Holarctic grey wolf.

The IUCN recognizes 12 Holarctic grey wolf subspecies with reference to Nowak [4], who historically listed the subspecies *C. l. campestris* for the northern parts of Central Asia*,* replaced by *C. l. chanco* in southern adjacent regions in Central Asia. These Central Asian subspecies *C. l. campestris*, *C. l. chanco* as well as *C. l. desertorum* were later pooled with *C. l. lupus* by Sillero-Zubiri *et al*. [3].

Holarctic grey wolf taxonomy is the subject of an ongoing debate due to continuing novel genetic insights across the species' range [6–8,10,17–19]. This study identifies the wolves in the Himalayas of northwestern Nepal as belonging to the previously discovered Himalayan wolf clade as described through molecular analysis of mtDNA [6,7]. We confirm the distinction in these wolves with mtDNA D-loop and cytochrome *b* sequences and for the first time using also X- and Y-chromosome sequences, and assess the phylogeny of this wolf lineage by putting it in context with canid molecular data from around the globe. In addition, this study summarizes the currently available genomic evidence around the Himalayan wolf lineage with geographic origin to support clarification of its taxonomy and distribution range.

## 2. Material and methods

### 2.1. Study area

**Figure 1.**
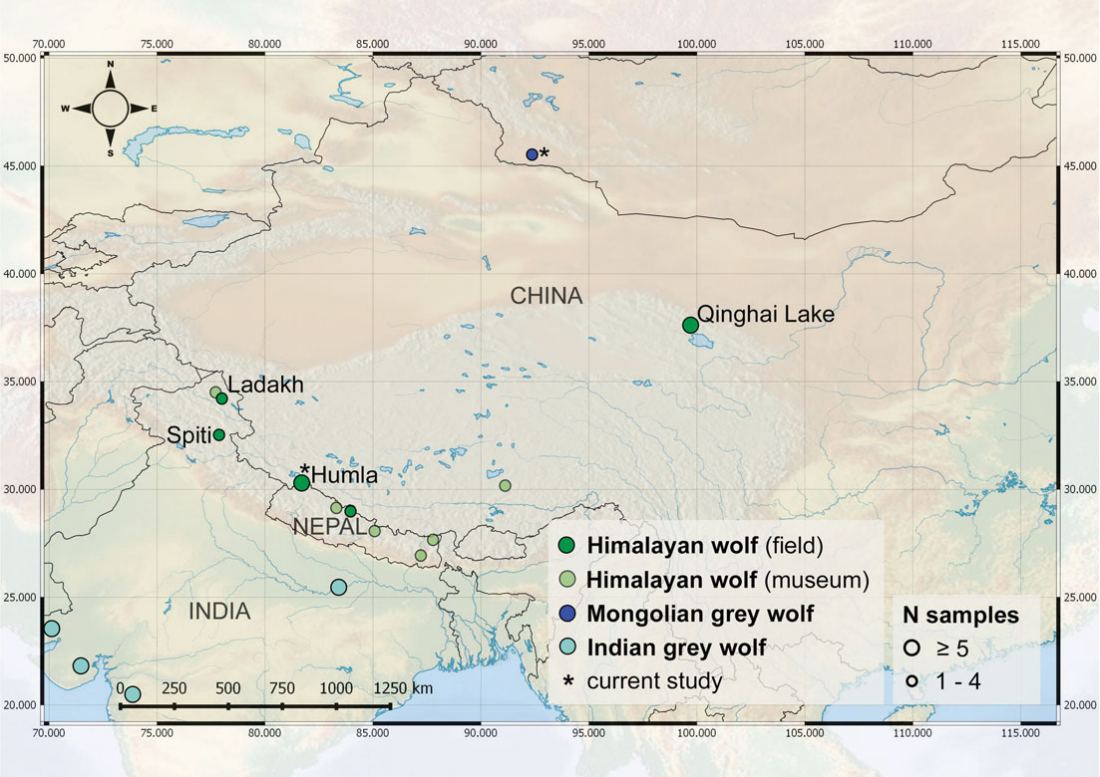
Overview of the current genetic evidence of the Himalayan wolf distribution. The data shown originates from the current and previous studies (Himalayan wolf field collected samples, dark green; Himalayan wolf museum specimens, light green). For overview, samples originating from Holarctic grey wolf lineages found in the region are also shown, i.e. Mongolian grey wolf *Canis lupus chanco* (dark blue) and Indian grey wolf *Canis lupus pallipes* (light blue). This study generated 72 Himalayan wolf sequences from 104 field collected samples in Humla, Nepal. The additional data shown derive from other studies [6,7,13].

The field study was conducted during July–August 2015 in the northwestern district of Humla in Nepal (figure 1). The study area of approximately 384 km^2^ (29.97°–30.36° N, 81.50°–82.05° E) is situated in Limi Village Development Committee and covers elevations from 3700 to 5390 m.a.s.l. The study area is situated in arid zones of the Himalayas [20] and characterized by alpine steppe vegetation. The area contains no permanent human settlements, but it is seasonally used by nomadic pastoralists to herd yaks, horses, sheep and goats during summer.

### 2.2. Collection of genetic material

A total of 104 samples from putative wolf scat and hair were collected along a total transect length of 605 km. The majority of transects consisted of opportunistic searches of the study area, and included also systematic prey distance sampling transects of two 1.5 km transects per 4 × 4 km grid cell. Ridge lines, streams and valley floors are topographic features known to be preferred travelling routes for wolves and were extensively searched [21].

Scats were swabbed and stored in Isohelix solution (Isohelix Ltd) and hair was stored with silica desiccant in a paper envelope. For genetic sampling, the faecal surface was scrubbed with a swab and then rinsed in the Isohelix solution, with this process being repeated two to three times. The swabbing was done on the outer shiny layer of the tapered end of the wolf scat. Scat age classification was adapted from Jackson & Hunter [22] and only recent to fresh wolf scats were sampled. The samples were kept out of sunlight in fresh temperatures until transfer to the genetic laboratory where they were stored in a freezer at −20°C. All further genetic laboratory protocols can be found in the electronic supplementary material.

### 2.3. Phylogenetic analysis

Sequences generated from Nepal were compared with canid reference sequences from around the globe obtained from the NCBI GenBank database (electronic supplementary material, tables S2 and S3). We constructed phylogenies using Bayesian and maximum-likelihood methods for D-loop and cytochrome *b* sequences. The dataset analysed consisted of 72 canid D-loop sequences from this study collected in Nepal and 148 canid D-loop reference sequences from NCBI GenBank. For cytochrome *b*, the dataset consisted of 24 sequences from this study collected in Nepal and 104 reference sequences from NCBI GenBank. Sequences were aligned using the ClustalW algorithm implemented in Geneious v. 8.1.8 to a consensus length of 242 bp for D-loop and of 508 bp for cytochrome *b* with final corrections done by eye. Phylogenies were built with MrBayes [23] with 11 002 tree building iterations (D-loop: chainlength 1 100 000, subsample frequency 200, burn-in-length 110 000, samples analysed 4951; cytochrome *b*: chainlength 1 100 000, subsample frequency 200, burn-in-length 110 000, samples analysed 4951), and maximum-likelihood phylogenies were built with PAUP* [24] (100 bootstrapping replications). The phylogenies were rooted with red fox (*Vulpes vulpes,* Linnaeus, 1758) sequences collected in the study area (NCBI GenBank accessions KY996531 and KY996535). Additional phylogenies built with Neighbour-joining and Tamura-Nei models of genetic distance showed the same arrangement and are found in the electronic supplementary material, figures S1 and S2. Haplotype networks were drawn with the software tool PopART (http://popart.otago.ac.nz) using Median-joining and TCS networks [25]. Evolutionary divergence between the Himalayan wolf, Holarctic grey wolf, African wolf, golden jackal, coyote and red fox was calculated with the software MEGA using the maximum composite likelihood distance between groups of nucleotide sequences [26,27]. For a subset of high-quality samples from the study area in Humla (Nepal), final intron sequences of the zinc-finger X-chromosomal (ZFX) and Y-chromosomal (ZFY) genes were generated following protocols described elsewhere [8,28–30]. For the primer sequences used for the zinc-finger protein gene analysis, see the electronic supplementary material, table S4. To test the method and verify our results, samples of African wolf [31] and Holarctic grey wolf were also sequenced at both these zinc-finger protein genes. Owing to the long PCR fragments involved (greater than 834 bp) and inevitable DNA degradation in non-invasive samples, it was only possible to obtain high-quality sequences of the sex genes for a small subset of the samples: five complete and one partial sequence for the Y-chromosome, and nine complete sequences for the X-chromosome.

## 3. Results

**Figure 2.**
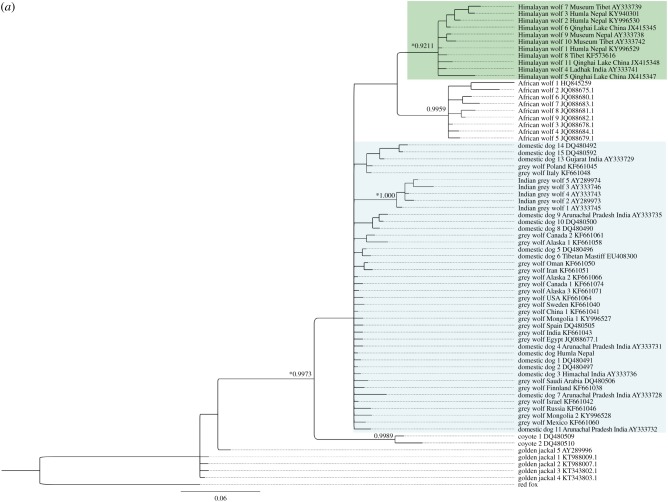

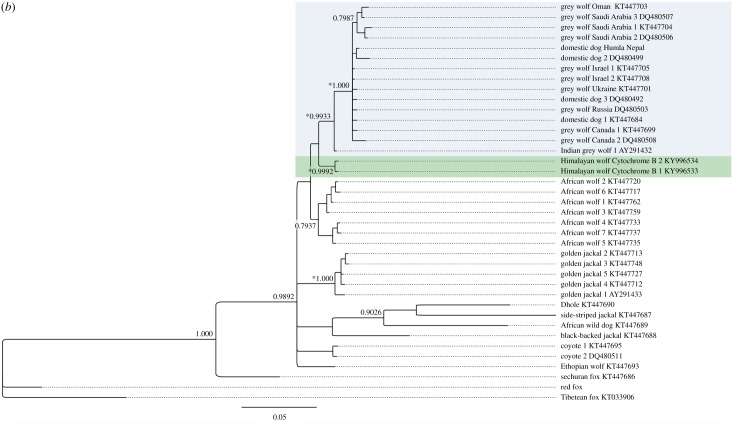
Bayesian phylogeny based on (*a*) 242 bp D-loop mtDNA sequences and (*b*) 508 bp cytochrome *b* mtDNA sequences with Bayesian posterior probability values at nodes. Asterisks at nodes indicate greater than or equal to 80% bootstrap support based on maximum-likelihood analyses and greater than or equal to 0.90 posterior probability from Bayesian inference. The Himalayan wolf (green) is a distinct monophyletic clade relative to the Holarctic grey wolf clade (blue) which also includes domestic dogs. Haplotypes without accession numbers are sequences generated in this study (electronic supplementary material, table S1).

We found that the Himalayan wolf forms a distinct monophyletic clade to the Holarctic grey wolf, supported by both the D-loop mtDNA and the cytochrome *b* mtDNA phylogeny (figure 2*a,b*), similar to previous studies by Sharma *et al*. [6] and Aggarwal *et al*. [7]. In addition, the results from the zinc-finger protein gene sequences at the Y-chromosome (1176 bp) and the X-chromosomes (514 bp) independently support these findings from the mtDNA data.

**Figure 3.**
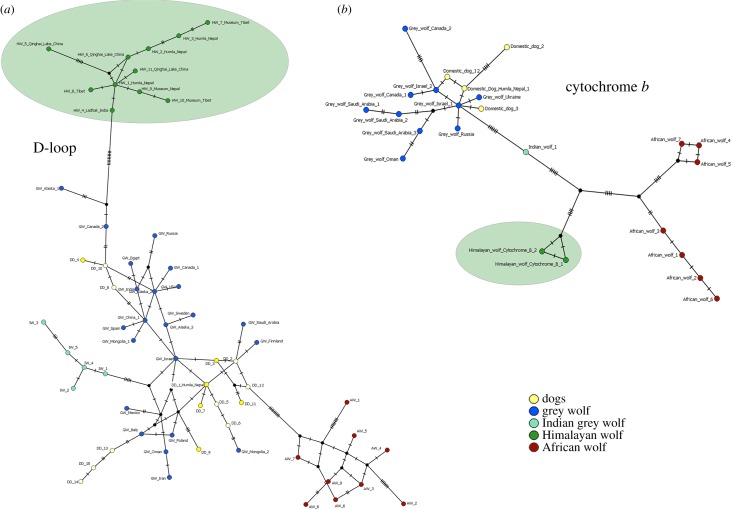
Median-joining networks based on (*a*) 242 bp D-loop haplotypes of *Canis* species and (*b*) 508 bp cytochrome *b* haplotypes of *Canis* species. Himalayan wolves (HW, green), African wolves (AW, red) form monophyletic clades. The grey wolf complex clusters with grey wolves from around the globe (GW, blue) including Indian grey wolves (IW, light blue) and domestic dogs (DD, yellow).

We found 76 unique canid D-loop haplotypes in the analysed dataset. Of these, 62 were wolves (including Holarctic grey wolf *C. lupus* spp., African wolf *C. aureus lupaster* and Himalayan wolf *C. himalayensis*), while the other 14 canid haplotypes of dhole (*Cuon alpinus,* Pallas, 1811), African wild dog (*Lycaon pictus,* Temminck, 1820), coyote, golden jackal (*C. aureus*, Linnaeus, 1758), side-striped jackal (*Canis adustus,* Sundevall, 1847) and red fox were used to put the collected data in a wider canid phylogenetic context. The Himalayan wolf clade contained 11 unique D-loop haplotypes (figure 2*a*), of which three haplotypes were found in the study area in Humla (Nepal): ‘Himalayan wolf haplotype 1’ (13 samples, NCBI GenBank accession KY996529), ‘Himalayan wolf haplotype 2’ (56 samples, NCBI GenBank accession KY996530) and ‘Himalayan wolf haplotype 3’ (seven samples, NCBI GenBank accession KY940301). The complete haplotype dataset used in the analysis with accession numbers is found in the electronic supplementary material, tables S1–S3. The monophyletic Himalayan wolf clade is supported with posterior probability/maximum-likelihood bootstrap of greater than 0.92/85 values for D-loop and posterior probability/maximum-likelihood bootstrap greater than 0.99/93 values for cytochrome *b* (figure 2*a,b*). Bayesian and neighbourhood-joining phylogenies based on D-loop and cytochrome *b* sequences showed the same structure (figure 2; electronic supplementary material, figure S2). Furthermore, the Himalayan wolf clade was supported similarly in both Median-joining and TCS haplotype networks (figure 3*a,b*).

Two Nepalese Himalayan wolf cytochrome *b* haplotypes were found in the samples from the study area in Humla (Nepal) which differed from Holarctic grey wolf haplotypes with 14 transitions and two transversions in the nucleotide sequence. For D-loop, the nucleotide differences between Holarctic grey wolf and the haplotype ‘Himalayan wolf D-loop 1’ were 12 transitions, 14 transitions for haplotype ‘Himalayan wolf D-loop 2’ and 16 transitions for the haplotype ‘Himalayan wolf D-loop 3’.

Another unique canid haplotype was found in three samples collected in Humla (Nepal), of which two samples were deliberately collected as domestic dog. From this putative domestic dog mitochondrial genome, we found one unique D-loop ‘Domestic dog D-loop Nepal 1’ (NCBI GenBank accession KY996526) and one unique cytochrome *b* haplotype ‘Domestic dog Cytochrome B 1’ (NCBI GenBank accession KY996532). This dog haplotype clusters with domestic dog *C. lupus familiaris* and Holarctic grey wolf *C. lupus* spp. haplotypes from around the globe in both D-loop and cytochrome *b* phylogenies (figure 2*a,b*).

The D-loop haplotype ‘Himalayan wolf D-loop 1’ found in Humla (Nepal) is identical to Himalayan wolf sequences deposited on NCBI GenBank by Sharma *et al*. [6] and Aggarwal *et al*. [7] (electronic supplementary material, tables S2 and S3; figure 2*a*). These sequences used by Aggarwal *et al*. [7] identical to ‘Himalayan wolf D-loop 1’ derive from captive animals in the Padmaja Naidu Zoo in India which descend from two to three wild captured wolves caught in the Trans-Himalayan region without a more specific capture location available [7,32]. The sequences used by Sharma *et al*. [6] identical to ‘Himalayan wolf D-loop 1’ originate from a captive individual caught in Spiti Valley (Himachal Pradesh, India), approximately 400 km northwest from the study area in Humla (Nepal).

Our analysis indicates that the Himalayan wolf lineage might be found as far north as Qinghai Lake in Qinghai Province in the People's Republic of China. This is indicated by our finding that unpublished D-loop sequences originating from Qinghai Lake on the Tibetan Plateau in the People's Republic of China and derived from NCBI GenBank were identical to Himalayan wolf D-loop haplotypes found in Nepal in this study (no further information could be obtained). These sequences from Qinghai Lake were designated as *C. lupus chanco* but matched with the haplotypes found in Nepal as follows: i.e. ‘Himalayan wolf D-loop 1’ identical with JX415352 and JX415350; ‘Himalayan wolf D-loop 2’ identical with JX415351; ‘Himalayan wolf D-loop 3’ identical with JX415343. In addition, other samples originating from Qinghai Lake and retrieved from NCBI GenBank present three additional unique haplotypes within the Himalayan wolf clade that were not found in Humla (Nepal) (i.e. JX415348, JX415345 and JX415347).

The understandable confusion around wolf scientific naming in this region is apparent from sequences on NCBI GenBank coming from wider locations on the Tibetan Plateau and attributed to different scientific names, i.e. either *C. l. chanco or C. l. laniger*, which all do cluster within the Himalayan wolf clade in this study (e.g. *C. l. chanco* NCBI GenBank accessions*:* AY333738, AY333739, AY333740, AY333741, AY333742, JX415343, JX415344, JX415345, JX415347, JX415348, JX415350, JX415351 and JX415352; *C. lupus laniger* NCBI GenBank accession*:* KF573616).

By contrast, Mongolian grey wolf (*C. l. chanco*) samples from individuals in the Zürich Zoo and originating from the Great Gobi B in Mongolia (R Zingg 2016, personal communication) clustered within the Holarctic grey wolf clade (figure 2*a*). It is currently uncertain based on the data we present here whether *C. l. chanco* should be considered as a distinct group within the Holarctic grey wolf as *C. l. chanco* samples do not seem to form a monophyletic clade.

Evolutionary divergence estimates between the D-loop sequences show a slightly greater distance between Holarctic grey wolf/Himalayan wolf (0.069) than between Holarctic grey wolf/African wolf (0.066), while the cytochrome *b* sequences show a greater distance between Holarctic grey wolf/African wolf (0.044) than between Holarctic grey wolf/Himalayan wolf (0.038) (table 2).

**Table 2.** Evolutionary distances (maximum composite likelihood analysis with MEGA) between Himalayan wolf, African wolf, Holarctic grey wolf, golden jackal, coyote and red fox for D-loop and cytochrome *b* mtDNA sequences.

	Holarctic grey wolf		Himalayan wolf		African wolf		golden jackal		coyote	
	D-loop	cyt *b*	D-loop	cyt *b*	D-loop	cyt *b*	D-loop	cyt *b*	D-loop	cyt *b*
Himalayan wolf	0.069	0.038								
African wolf	0.066	0.044	0.062	0.028						
golden jackal	0.118	0.076	0.127	0.052	0.136	0.051				
coyote	0.099	0.062	0.128	0.050	0.103	0.050	0.124	0.056		
red fox	0.328	0.179	0.366	0.176	0.332	0.175	0.300	0.180	0.341	0.170

**Figure 4.**
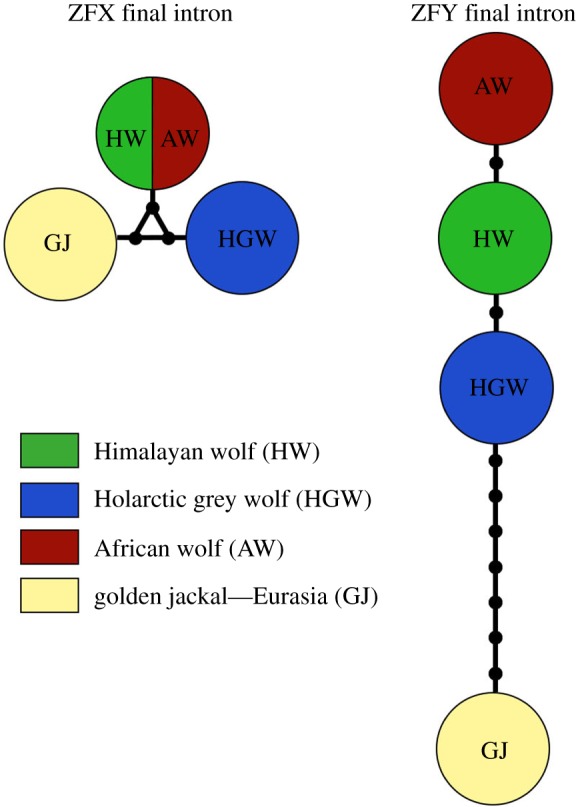
Haplotype network showing the ZFY and ZFX final intron sequences of Himalayan wolf (green), African wolf (red), Holarctic grey wolf (blue) and golden jackal (red). The black dots on the internode represent indels and substitutions between the haplotypes.

One unique haplotype for each the ZFY and the ZFX sequence was found in the Himalayan wolf samples collected in the study population in Humla (NCBI GenBank accessions: MF101862 and MF101863). For the Holarctic grey wolf and African wolf samples, we found the same ZFY/ZFX haplotypes as in Koepfli *et al.* [8]. Comparing the results of the ZFY final intron sequence among Himalayan, Holarctic grey and African wolf supports that the Himalayan wolf forms a distinct wolf lineage. The Himalayan wolf ZFY is different from the Holarctic grey wolf at position 1010, where both Himalayan and African wolf share the nucleic acid T, rather than the G found in Holarctic grey wolf. A 30 bp indel shared by both Himalayan and Holarctic grey wolf is not found in the African wolf (table 3 and figure 4). For the ZFX final intron sequence, we found identical haplotypes for both the Himalayan wolf and the African wolf from Kenya. By contrast, the Holarctic grey wolf, including domestic dogs, shows a haplotype different at two positions from the Himalayan wolf and the African wolf (indel at position 328, substitution at 425; table 4).

**Table 3.** Informative positions found in the final intron sequences of the zinc-finger Y-chromosomal (ZFY, 1176 bp) results of Himalayan wolf, compared with African wolf, Holarctic grey wolf, golden jackal and coyote. Accession numbers for NCBI GenBank reference samples used in the analysis are provided.

			ZFY final intron									
			23	123	193–201	236–446	880–909	1010	1036–1037	1056	1094	
seq. source	species	*n*	A/G	1 bp insertion	9 bp insertion	210 bp SINEII insertion	30 bp deletion	T/G	2 bp insertion	G/A	T/C	haplotype
NCBI GenBank	coyote *C. latrans* (JN663892.1)	1	A	—	—	—	30 bp	T	—	G	C	E
	Holarctic grey wolf *C. lupus* Canada (KT448254.1)	1	A	—	—	—	30 bp	G	—	G	T	C
	golden jackal *C. aureus* Serbia (KT448259.1)	1	G	A	9 bp	210 bp	—	T	TA	A	T	A
	golden jackal *C. aureus* Israel (KT448266.1)	1	G	A	9 bp	210 bp	—	T	TA	A	T	
	African wolf *C. aureus lupaster* Kenya and Morocco (KT448270.1, KT448267.1)	2	A	—	—	—	—	T	—	G	T	B
this study	African wolf *C. aureus lupaster* Morocco	1	A	—	—	—	—	T	—	G	T	
	Himalayan wolf *C. I. himalayensis* Nepal (MF101862)	5 and partial	A	—	—	—	30 bp	T	—	G	T	D
	Holarctic grey wolf *C. lupus* Europe and Mongolia (captive Zurich Zoo)	2	A	—	—	—	30 bp	G	—	G	T	C

**Table 4.** Informative positions found in the final intron sequences of the ZFX (514 bp) results of Himalayan wolf, compared with African wolf, Holarctic grey wolf and golden jackal. NCBI GenBank accession numbers are provided.

			ZFX final intron			
			328	381	425	
seq. source	species	*n*	1 bp insertion	T/A	A/G	haplotype
NCBI GenBank	golden jackal *C. aureus* Israel (KT448243.1)	1	G	A	A	A
	African wolf *C. aureus lupaster* Kenya (KT448251.1)	1	G	T	G	B
	Holarctic grey wolf *C. lupus* Canada (KT448225.1)	1	—	T	A	C
this study	Holarctic grey wolf *C. lupus* Europe	1	—	T	A	C
	Holarctic grey wolf *C. lupus* Mongolia	4	—	T	A	
	Holarctic grey wolf *C. lupus* Arabian	1	—	T	A	
	dog *C. lupus* Nepal	2	—	T	A	
	Himalayan wolf *C. I. himalayensis* Nepal (MF101863)	9	G	T	G	B

## 4. Discussion

This study provides genetic evidence in support of the distinct Himalayan wolf lineage found in Central Asia, the formal taxonomic recognition of which is pending due to limited data from contemporary wild populations. Our results confirm findings of previous studies from the broader Himalayan region, largely based on museum specimens and zoo animals [6,7,13,33], which place the Himalayan wolf as a distinct monophyletic lineage relative to the Holarctic grey wolf. This study expands the existing data on the Himalayan wolf with sampling of a contemporary living wolf population in a previously unconfirmed location in northwestern Nepal. Further, this study updates the currently limited understanding of the distribution range of the Himalayan wolf by integrating available genetic and geographic data from previous studies.

We show the genetic distinctness between the Himalayan wolf and the Holarctic grey wolf based on 242 bp D-loop mtDNA and 508 bp cytochrome *b* mtDNA sequences. Further, the analyses of the X- and Y-linked zinc-finger protein gene (ZFX: 514 bp and ZFY: 1176 bp) sequences support the results emerging from the mtDNA (figure 4). Hence, multiple independent pieces of genomic evidence indicate that the Himalayan wolf forms a distinct monophyletic wolf lineage to the Holarctic grey wolf similar to the recently posited African wolf. At the mtDNA genes tested here, the level of divergence from the Holarctic grey wolf to the Himalayan wolf is similar to that of the African wolf (table 2). Within the Himalayan wolf population in our study area in Humla (Nepal), we found one haplotype for each of the X- and Y-linked zinc-finger protein gene (ZFX and ZFY) sequences. Fewer haplotypes are expected at this gene sequence, as the sex chromosomes are a slow evolving area, and a similar situation was found for the African wolf (tables 3 and 4) [8]. The Himalayan wolf emerges as a basal distinct monophyletic wolf lineage to the Holarctic grey wolf in phylogenies derived from cytochrome *b*, an arrangement which is additionally supported by the intermediate placement of the Himalayan wolf between the African wolf and Holarctic grey wolf at ZFY and the sharing of a ZFX haplotype with the African wolf. At D-loop, the Himalayan wolf forms a distinct monophyletic clade within the Holarctic grey wolf clade (figure 2*a,b* and tables 3 and 4), but it does not occupy a basal position with respect to the Holarctic grey wolf. Sequencing of longer fragments of D-loop (currently 242 bp) may resolve this conflict.

The Himalayan wolf lineage is more divergent from the Holarctic grey wolf than the IUCN recognized Indian grey wolf subspecies (*C. lupus pallipes*), which forms a monophyletic clade nested within the Holarctic grey wolf complex [6,10]. Thus, in this context, the current and previous studies suggest the need for adjusting the taxonomy of the Himalayan wolf in recognition of its genetic uniqueness but also imply more generally the need for revision of wolf subspecies and units of conservation concern.

Based on its clear phylogenetic distinction and older age of divergence relative to the Holarctic grey wolf and for consistency within the existing IUCN naming system, the Himalayan wolf merits at minimum classification at subspecies level of special conservation concern (i.e. *C. lupus himalayensis;* and possibly *C. himalayensis* as proposed by Aggarwal *et al*. [7]). Future assessments of more nuclear data, and critically the factors causing reproductive isolation (if they indeed exist) might then further validate the taxon's status as a full species (i.e. *C. himalayensis*).

Our findings show that wolf individuals from the Tibetan Plateau and the Himalayan region, which had historically been assigned to *C. l. chanco* or *C. l. laniger*, fall within the Himalayan wolf lineage, while wolf individuals from Mongolia, also assigned as *C. l. chanco*, phylogenetically group within the Holarctic grey wolf complex. Thus, the subspecies name *C. l. chanco* (often attributed the common name ‘Mongolian grey wolf’) seems accurately used for grey wolves found in northern parts of Central Asia such as Mongolia. However, the justification for its own subspecies *C. l. chanco* is limited considering the data here presented, as the ‘accurately’ named *C. l. chanco* sequences group within the monophyletic Holarctic grey wolf clade, but without forming an own distinct monophyletic group within that.

The Mongolian grey wolf and the Himalayan wolf seem to be found in different parts of Central Asia. The currently available data indicate that the distribution of the Himalayan wolf lineage extends west of Kashmir valley (India) across the Himalayan Mountains, with samples from wild individuals available from Kashmir valley (India) [6], Spiti Valley and Ladakh (India) [6,7], Humla (Nepal) (this study) and the Annapurna Conservation Area in Mustang (Nepal) [13]. An advanced understanding of the Himalayan wolf distribution range derives from including all genetic data from the region in our analysis as available on NCBI GenBank. Our analysis suggests that the range of the Himalayan wolf lineage extends north from the Himalayas across the Tibetan Plateau as far as Qinghai Lakes in Qinghai Province in the People's Republic of China, while the Mongolian grey wolf (*C. l. chanco—*if it can be considered as such, see above) is found in Mongolia. This raises questions about the distribution boundaries between the Himalayan wolf and the grey wolf in Mongolia, and also what evolutionary processes might be maintaining them.

**Figure 5.**
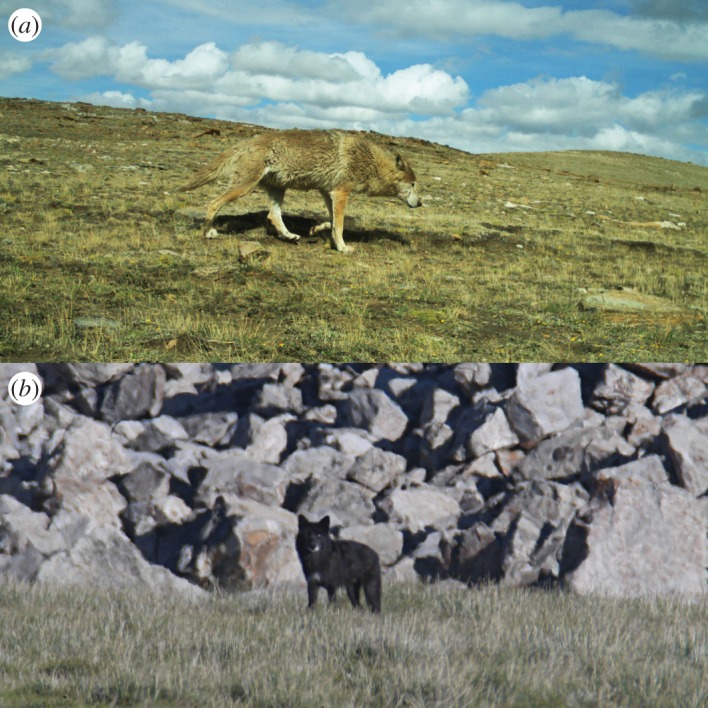
Himalayan wolf adults in Humla, Nepal. Photograph (*a*) shows a pale-coloured wolf individual, and (*b*) a black-coloured wolf individual (© Geraldine Werhahn).

Historically, the naming of wolves in the Himalayan region has been based on scattered observations. Shrotryia *et al.* [1] include a historical overview of the scientific naming of wolves in this region briefly summarized in the following: Hodgson [34] provided a first description of what seemed a Himalayan wolf and attributed it to *C. laniger*. Blanford [35] then merged this taxon with *C. lupus*. They described the wolf variety found in Tibet and Ladakh as pale coloured with woolly underfur and also mentioned black individuals [1]—both morphologies were frequently observed in the study area in the Himalayas of Nepal (figure 5). Later, Pocock [36] described *C. laniger* as a *C. lupus* subspecies and combined it with the more widely distributed *C. lupus chanco.* The voucher for the genetic sequence HW9_Museum Nepal (figures 2*a* and 3*a*) is a museum specimen in the Natural History Museum in South Kensington (UK) from the collection by B. Hodgson from Nepal (NCBI GenBank accession AY333738; BM58.6.24.61) [6]. The voucher for the genetic sequence HW7_Museum Tibet (figures 2*a* and 3*a*) is a museum specimen in the Natural History Museum in South Kensington (UK) collected in Tibet by A.H. Savage-Landor and described as a black animal (NCBI GenBank accession AY333739; BM99.12.29.1) [6].

### 4.1. Applying species concepts

The contemporary recognized Holarctic grey wolf subspecies show no reciprocally monophyletic clades, with the exception of the Himalayan wolf and Indian grey wolf, likely due to glaciation and dispersal events in the past [11,37]. Leading up to its divergence, the Himalayan wolf seems to have formed an independently evolving metapopulation lineage based on the mitochondrial data, which is a primary criterion for defining species status under the unified species concept [38]. If we were to define taxa according to the genetic and phylogenetic species concept, this monophyletic lineage showing 3.8% divergence from Holarctic grey wolf (table 2) could be used to justify that the Himalayan wolf is a distinct species. Studies on other mammalian taxa [39] conclude that greater than 2–11% divergence in cytochrome *b* is indicative of conspecific populations and possibly valid species, and therefore merits additional studies concerning species status in these cases. However, at least 26 different species concepts exist [40]. Inherent to them all is that a species is a cohesive cluster of individuals representing a different lineage as a result of at least partially different evolutionary paths. Species concepts suitable also for conservation should take fitness into account, and as such, the species concepts considered above are not generally thought to be the most suitable for making conservation-relevant decisions about taxonomy. The ideal species concept for conservation purposes maximizes benefits for the species in terms of reproductive fitness, sustains evolutionary adaptation processes and facilitates conservation. The Himalayan wolf, as indicated in the current and previous studies, fulfils the criteria of the phylogenetic, the evolutionary and the unified species concepts [40]. To test the biological species concept and differential fitness species concept which are more appropriate for conservation purposes [40], more nuclear genomic data, including in best case also functional genes, from across the Himalayan wolf's range should be analysed. Such data could then also be used to assess geographic range boundaries with the Holarctic grey wolf, and evaluate the evolutionary processes that maintain these species boundaries. Other fitness relevant data in the form of biogeographic, phenotypic and karyotypic evidence might further add to the argument if these become available. Investigation into whether hybridization with the domestic dog is occurring may also be of conservation concern, although it may prove hard to disentangle from introgression from the Holarctic grey wolf as this may also occur.

Given the mounting genetic evidence surrounding the Himalayan wolf and African wolf lineage, it seems inevitable that a wider revision of canid taxonomy on the Eurasian continent and North Africa may be required in due course.

## 5. Conclusion

We present genetic evidence for the Himalayan wolf lineage collected from a contemporary wild wolf population in northwestern Nepal. Our study adds to the growing evidence around the evolutionarily distinct Himalayan wolf with substantial field collected genetic data combined with genetic and geographic data from previous studies, and is the first to include evidence from sex chromosomes. The genetic evidence (mtDNA cytochrome *b*, mtDNA D-loop and sex chromosomes) point to a distinct monophyletic position of the Himalayan wolf with respect to the Holarctic grey wolf. We infer from this and previous studies that the Himalayan wolf lineage deserves taxonomic recognition at subspecies level (i.e. *C. lupus himalayensis*). Given further research, especially involving nuclear DNA, elevation to *C. himalayensis* as proposed by Aggarwal *et al*. [7] may be justified. Adjusting the taxonomy of the Himalayan wolf to its phylogeny will not only adequately reflect its genetic distinctness, but is also essential to advance future research into the genetics, ecology and conservation of the Himalayan wolf.

## Supplementary Material

SUPPLEMENTARY MATERIAL; Cytochrome B Haplotypes; D-loop Haplotypes

## Data accessibility

All sequence data supporting this publication are deposited on NCBI GenBank under the accessions (KY996526-KY996535, KY94030, MF101862 and MF101863). All accession numbers are included in the text and figures of the main article, and tables of the electronic supplementary material. The electronic supplementary material accompanying this article contains details of the genetic analysis method, additional figures and a complete list of all data used in the phylogenetic analyses with accession numbers and references.
