# Supplementary material for: Phylogenetic evidence for the ancient Himalayan wolf: towards a clarification of its taxonomic status based on genetic sampling from western Nepal
Source: R Soc Open Sci. 2017 Jun 7;4(6):170186. doi: 10.1098/rsos.170186 (PMC5493914; doi:10.1098/rsos.170186)
Supplement: SUPPLEMENTARY MATERIAL; Cytochrome B Haplotypes; D-loop Haplotypes [file rsos170186supp1.docx]

**SUPPLEMENTARY MATERIAL**

**METHODS**

***Genetic analysis procedure***

**DNA extraction**

A mixture of commercially available Isohelix and Qiagen Stool kit parts were used for DNA extraction. Samples were centrifuged for one minute at 4000rpm followed by pipetting the supernatant into clean 1.5ml flip-top tube (~450μl). 250μl InhibitEX solution was added after which it was vortexed for a minute. Then the sample solution was left for one minute at room temperature to allow the inhibitors to be absorbed. This was followed by centrifugation for one minute at full speed (14.5x1000rpm) to pellet remaining stool particles. The supernatant was then pipetted into a 1.5ml flip-top tube and 25μl of proteinase K solution was added. This was placed on a thermoblock at 60˚C, and mixed for approximately one hour. Then 500μl CT solution was added and the tube inverted several times to mix, followed by briefly centrifuging to remove liquid from the lid. Of this 600µl was pipetted into a spin column collection tube and centrifuged for one minute at full speed. This was repeated with new collection tubes until all the lysate was through the spin column. The spin column was then put into a clean collection tube and spun through 500µ Qiagen l AW1 wash buffer for one minute at full speed. The spin column was then put into a clean collection tube and spun through 500µl Qiagen AW2 wash buffer for one minute at full speed. The spin column was then put into a clean collection tube and spun for two minutes at 14.5x1000rpm to remove any residual ethanol from the cartridge. Then it was transferred to a labelled 1.5ml flip-top tube and 75μl AE elution buffer, which was heated to ~55˚C on the thermoblock. It was then pipetted on to the cartridge and left to settle for one minute followed by spinning for one minute at full speed. The DNA sample was then quantified and stored at -20°C.

**DNA sequencing**

Cytochrome *b* and D-loop sequences of the mtDNA, and X- and Y-linked zinc-finger protein gene (ZFX and ZFY) sequences were generated from 104 samples collected in 2015.

Primers for both D-loop and cytochrome *b* sequences were designed at RZSS WildGenes laboratory. For the X- and Y-linked zinc-finger protein gene (ZFX and ZFY) sequences were amplified using the protocol and primers used by (1–3) (for the primer sequences see supplementary material Table S4).

The D-loop control region primers amplified a 296bp long section (forward primer WolfJack dloop: GCACCCAAAGCTGAAATTCT, reverse primer WolfJack dloop: ATGGGCCCGGAGCGAGAAGAG). Sequencing primers were diluted to 10µM and Taq master mix (Thermo Fisher Scientific Inc.) and were run with the following PCR program: Amplification performed with an initial denaturation step of 95˚C for 5 minutes, followed with 40 cycles for 30 seconds at 95˚C (denaturation), 61˚C for 30 seconds (primer annealing) and 72˚C for 60 seconds (elongation), and ending with 72˚C extension for 10 minutes. Samples were viewed on a 1.5% agarose gel to check if they have amplified.

For cytochrome *b*, primers amplifying at the 353bp section were used (forward primer WolfJackCytb1: TTGTATTTCAACTATAAGAACAT, reverse primer WolfJackCytb: GCAAAGAATCGTGTTAGGGTTG).

The amplification procedure for the cytochrome *b* was as for the D-loop but with the following PCR thermocycling conditions: 95˚C for 5 minutes, then 35 cycles of 95˚C for 30 seconds, then 50˚C for 90 seconds and 72˚C for 30 seconds, followed by 60˚C for 30 minutes. As positive controls, 8 samples were repeated with D-loop forward, 27 with D-loop reverse, and 33 were repeated for both forward and reverse cytochrome *b*.

**Table S1.** Haplotypes of Himalayan wolves and dogs found in the study area in Humla (Nepal) with NCBI GenBank accessions.

| **Region** | **Species** | **Haplotype Name** | **GenBank Accession** |
| --- | --- | --- | --- |
| D-loop | *C. himalayensis* | Himalayan wolf D-loop 1 | KY996529 |
| D-loop | *C. himalayensis* | Himalayan wolf D-loop 2 | KY996530 |
| D-loop | *C. himalayensis* | Himalayan wolf D-loop 3 | KY940301 |
| D-loop | *C. lupus familiaris* | Domestic dog D-loop Nepal 1 | KY996526 |
| cytochrome *b* | *C. himalayensis* | Himalayan wolf Cytochrome B 1 | KY996533 |
| cytochrome *b* | *C. himalayensis* | Himalayan wolf Cytochrome B 2 | KY996534 |
| cytochrome *b*  ZFY  ZFX | *C. lupus familiaris*  *C. himalayensis*  *C. himalayensis* | Domestic dog Cytochrome B Nepal 1  Himalayan wolf Nepal ZFY  Himalayan wolf Nepal ZFX | KY996532  MF101862 MF101863 |

**Table S2.** Overview of the D-loop data used in the phylogenetic analysis. Listed are the new haplotypes of Himalayan wolf 1-3 and one domestic dog found in the study area in Humla (Nepal), and all reference sequences obtained from NCBI GenBank. Marked with an asterisk*** are sequences that are unpublished and have been directly submitted to NCBI GenBank by the respective authors. Reference sequences from the following publications were included: (3–13).

| **Haplotype** | **Name abbreviated** | **Unique Haplotypes Accession** | **Identical Sequences Accessions** | **Publication** |
| --- | --- | --- | --- | --- |
| Side-striped Jackal 1 |  | JQ088674.1 |  | Gaubert et al. (2012) |
| Side-striped Jackal 2 |  | JQ088669.1 |  | Gaubert et al. (2012) |
|  |  |  | JQ088670.1 | Gaubert et al. (2012) |
|  |  |  | JQ088671.1 | Gaubert et al. (2012) |
|  |  |  | JQ088672.1 | Gaubert et al. (2012) |
|  |  |  | JQ088673.1 | Gaubert et al. (2012) |
| African Wild Dog |  | KT448283.1 |  | Koepfli et al. (2015) |
| Asian Dhole |  | KT448282.1 |  | Koepfli et al. (2015) |
| Coyote 1 |  | DQ480509 |  | Björnerfeldt et al. (2006) |
|  |  |  | DQ480511 | Björnerfeldt et al. (2006) |
| Coyote 2 |  | DQ480510 |  | Björnerfeldt et al. (2006) |
| Red Fox |  | KY996531 |  | This study |
| Himalayan wolf 1 Humla Nepal | HW1_Humla Nepal | KY996529 |  | This study |
|  |  |  | AY333740 | Sharma et al. (2004) |
|  |  |  | AY289986 | Aggarwal et al. (2007) |
|  |  |  | AY289995 | Aggarwal et al. (2007) |
|  |  |  | AY289985 | Aggarwal et al. (2007) |
|  |  |  | AY289994 | Aggarwal et al. (2007) |
|  |  |  | AY289977 | Aggarwal et al. (2007) |
|  |  |  | AY289993 | Aggarwal et al. (2007) |
|  |  |  | AY289992 | Aggarwal et al. (2007) |
|  |  |  | AY289991 | Aggarwal et al. (2007) |
|  |  |  | AY289978 | Aggarwal et al. (2007) |
|  |  |  | AY289990 | Aggarwal et al. (2007) |
|  |  |  | AY289979 | Aggarwal et al. (2007) |
|  |  |  | AY289980 | Aggarwal et al. (2007) |
|  |  |  | AY289989 | Aggarwal et al. (2007) |
|  |  |  | AY289981 | Aggarwal et al. (2007) |
|  |  |  | AY289988 | Aggarwal et al. (2007) |
|  |  |  | AY289982 | Aggarwal et al. (2007) |
|  |  |  | AY289983 | Aggarwal et al. (2007) |
|  |  |  | AY289987 | Aggarwal et al. (2007) |
|  |  |  | JX415352 | unpublished* |
|  |  |  | JX415350 | unpublished* |
| Himalayan wolf 2 Humla Nepal | HW2_Humla Nepal | KY996530 |  | This study |
|  |  |  | JX415351 | unpublished* |
|  |  |  | EU442884.2 | unpublished* |
| Himalayan wolf 3 Humla Nepal | HW3_Humla Nepal | KY940301 |  | This study |
|  |  |  | JX415343 | unpublished* |
| Himalayan wolf 4 Ladhak India | HW4_Ladhak | AY333741 |  | Sharma et al. (2004) |
| Himalayan wolf 5 Qinghai Lake China | HW5_Qinghai_Lake_China | JX415347 |  | unpublished* |
| Himalayan wolf 6 Qinghai Lake China | HW6_Qinghai_Lake_China | JX415345 |  | unpublished* |
| Himalayan wolf 7 Museum Tibet | HW7_Museum Tibet | AY333739 |  | Sharma et al. (2004) |
| Himalayan wolf 8 Tibet | HW8_Tibet | KF573616 |  | unpublished* |
|  |  |  | AB480742 | Ishiguro et al. (2009) |
| Himalayan wolf 9 Museum Nepal | HW9_Museum Nepal | AY333738 |  | Sharma et al. (2004) |
|  |  |  | JX415344 | unpublished* |
| Himalayan wolf 10 Museum Tibet | HW10_Museum Tibet | AY333742 |  | Sharma et al. (2004) |
| Himalayan wolf 11 Qinghai Lake China | HW11_Qinghai_Lake_China | JX415348 |  | unpublished* |
| Indian grey wolf 1 | IW 1 | AY333745 |  | Sharma et al. (2004) |
| Indian grey wolf 2 | IW 2 | AY289973 |  | Aggarwal et al. (2007) |
| Indian grey wolf 3 | IW 3 | AY333746 |  | Sharma et al. (2004) |
| Indian grey wolf 4 | IW 4 | AY333743 |  | Sharma et al. (2004) |
| Indian grey wolf 5 | IW 5 | AY289974 |  | Aggarwal et al. (2007) |
|  |  |  | AY289975 | Aggarwal et al. (2007) |
|  |  |  | AY289976 | Aggarwal et al. (2007) |
|  |  |  | AY289984 | Aggarwal et al. (2007) |
|  |  |  | AY333746 | Sharma et al. (2004) |
|  |  |  | AY333744 | Sharma et al. (2004) |
| Grey wolf_China 1 | GW China 1 | KF661041 |  | Thalmann et al. (2013) |
|  |  |  | KF661053 | Thalmann et al. (2013) |
| Grey wolf_Mongolia 1 | GW Mongolia 1 | KY996527 |  | WildGenes collection |
| Grey wolf_Mongolia 2 | GW Mongolia 2 | KY996528 |  | WildGenes collection |
| Grey wolf_Russia | GW Russia | KF661046 |  | Thalmann et al. (2013) |
| Grey wolf_Poland | GW Poland | KF661045 |  | Thalmann et al. (2013) |
| Grey wolf_India | GW India | KF661043 |  | Thalmann et al. (2013) |
|  |  |  | KF661054 | Thalmann et al. (2013) |
| Grey wolf_Egypt | GW Egypt | JQ088677.1 |  | Gaubert et al. (2012) |
|  |  |  | KF661055 | Thalmann et al. (2013) |
| Grey wolf_Saudi Arabia | GW Saudi Arabia | DQ480506 |  | Björnerfeldt et al. (2006) |
| Grey wolf_Israel | GW Israel | KF661042 |  | Thalmann et al. (2013) |
|  |  |  | AY333733 | Sharma et al. (2004) |
| Grey wolf_Oman |  | KF661050 |  | Thalmann et al. (2013) |
| Grey wolf_Iran |  | KF661051 |  | Thalmann et al. (2013) |
| Grey wolf_Finnland | GW Finnland | KF661038 |  | Thalmann et al. (2013) |
|  |  |  | KF661039 | Thalmann et al. (2013) |
|  |  |  | DQ480503 | Björnerfeldt et al. (2006) |
| Grey wolf_Sweden | GW Sweden | KF661040 |  | Thalmann et al. (2013) |
|  |  |  | KF661044 | Thalmann et al. (2013) |
|  |  |  | KF661049 | Thalmann et al. (2013) |
|  |  |  | KF661052 | Thalmann et al. (2013) |
|  |  |  | DQ480504 | Björnerfeldt et al. (2006) |
| Grey wolf_Italy | GW Italy | KF661048 |  | Thalmann et al. (2013) |
| Grey wolf_Spain | GW Spain | DQ480505 |  | Björnerfeldt et al. (2006) |
| Grey wolf_USA | GW USA | KF661064 |  | Thalmann et al. (2013) |
|  |  |  | KF661068 | Thalmann et al. (2013) |
|  |  |  | KF661069 | Thalmann et al. (2013) |
|  |  |  | KF661072 | Thalmann et al. (2013) |
| Grey wolf_Canada 1 | GW Canada 1 | KF661074 |  | Thalmann et al. (2013) |
| Grey wolf_Canada 2 | GW Canada 2 | KF661061 |  | Thalmann et al. (2013) |
|  |  |  | KF661062 | Thalmann et al. (2013) |
|  |  |  | KF661063 | Thalmann et al. (2013) |
|  |  |  | KF661056 | Thalmann et al. (2013) |
|  |  |  | DQ480508 | Björnerfeldt et al. (2006) |
| Grey wolf_Alaska 1 | GW Alaska 1 | KF661058 |  | Thalmann et al. (2013) |
| Grey wolf_Alaska 2 | GW Alaska 2 | KF661066 |  | Thalmann et al. (2013) |
| Grey wolf_Alaska 3 | GW Alaska 3 | KF661071 |  | Thalmann et al. (2013) |
|  |  |  | KF661059 | Thalmann et al. (2013) |
|  |  |  | KF661073 | Thalmann et al. (2013) |
|  |  |  | KF661057 | Thalmann et al. (2013) |
| Grey wolf_Mexico | GW Mexico | KF661060 |  | Thalmann et al. (2013) |
|  |  |  | KF661065 | Thalmann et al. (2013) |
| Domestic dog 12 | DD 12 | DQ480491 |  | Björnerfeldt et al. (2006) |
| Domestic dog 2 | DD 2 | DQ480497 |  | Björnerfeldt et al. (2006) |
|  |  |  | KF661036 | Thalmann et al. (2013) |
|  |  |  | DQ480498 | Björnerfeldt et al. (2006) |
| Domestic dog 3 Himachal India | DD 3 | AY333736 |  | Sharma et al. (2004) |
|  |  |  | DQ480499 | Björnerfeldt et al. (2006) |
|  |  |  | KF661037 | Thalmann et al. (2013) |
| Domestic dog 4 Arunachal Pradesh India | DD 4 | AY333731 |  | Sharma et al. (2004) |
| Domestic dog 5 | DD 5 | DQ480496 |  | Björnerfeldt et al. (2006) |
| Domestic dog 6 Tibetan Mastiff | DD 6 | EU408300 |  | unpublished* |
| Domestic dog 7 Arunachal Pradesh India | DD 7 | AY333728 |  | Sharma et al. (2004) |
|  |  |  | DQ480493 | Björnerfeldt et al. (2006) |
|  |  |  | DQ480501 | Björnerfeldt et al. (2006) |
|  |  |  | AY333727 | Sharma et al. (2004) |
| Domestic dog 8 | DD 8 | DQ480490 |  | Björnerfeldt et al. (2006) |
| Domestic dog 9 Arunachal Pradesh | DD 9 | AY333735 |  | Sharma et al. (2004) |
| Domestic dog 10 | DD 10 | DQ480500 |  | Björnerfeldt et al. (2006) |
|  |  |  | KF661047 | Thalmann et al. (2013) |
|  |  |  | DQ480494 | Björnerfeldt et al. (2006) |
|  |  |  | AY333730 | Sharma et al. (2004) |
|  |  | KF661050 |  | Thalmann et al. (2013) |
|  |  |  | DQ480507 | Björnerfeldt et al. (2006) |
|  |  | KF661051 |  | Sharma et al. (2004) |
| Domestic dog 11 Arunachal Pradesh | DD 11 | AY333732 |  | Sharma et al. (2004) |
| Domestic dog Nepal 1 | DD 1 Nepal | KY996526 |  | This study |
|  |  |  | AY333737 | Sharma et al. (2004) |
|  |  |  | DQ480495 | Björnerfeldt et al. (2006) |
| Domestic dog 13 Gujarat India | DD 13 | AY333729 |  | Sharma et al. (2004) |
| Domestic dog 14 | DD 14 | DQ480492 |  | Björnerfeldt et al. (2006) |
| Domestic dog 15 | DD 15 | DQ480502 |  | Björnerfeldt et al. (2006) |
| African Wolf 1 | AW1 | HQ845259 |  | Rueness et al. (2011) |
| African Wolf 2 | AW2 | JQ088675.1 |  | Gaubert et al. (2012) |
|  |  |  | JQ088676.1 | Gaubert et al. (2012) |
| African Wolf 3 | AW 3 | JQ088678.1 |  | Gaubert et al. (2012) |
| African Wolf 4 | AW 4 | JQ088684.1 |  | Gaubert et al. (2012) |
| African Wolf 5 | AW 5 | JQ088679.1 |  | Gaubert et al. (2012) |
| African Wolf 6 | AW 6 | JQ088680.1 |  | Gaubert et al. (2012) |
|  |  |  | KM670012 | Waters et al. (2015) |
| African Wolf 7 | AW 7 | JQ088683.1 |  | Gaubert et al. (2012) |
| African Wolf 8 | AW 8 | JQ088681.1 |  | Gaubert et al. (2012) |
| African Wolf 9 | AW 9 | JQ088682.1 |  | Gaubert et al. (2012) |
| Ethopian Wolf 1 |  | AY551930.1 |  | Gottelli et al. (2004) |
| Ethopian Wolf 2 |  | KT448281.1 |  | Koepfli et al. (2015) |
| Golden Jackal 1 |  | KT988009.1 |  | İbiş et al. (2015) |
| Golden Jackal 2 |  | KT988007.1 |  | İbiş et al. (2015) |
| Golden Jackal 3 |  | KT343802.1 |  | İbiş et al. (2015) |
|  |  |  | KT988006.1 | İbiş et al. (2015) |
| Golden Jackal 4 |  | KT343803.1 |  | İbiş et al. (2015) |
|  |  |  | HQ845260 | Rueness et al. (2011) |
|  |  |  | KT268319.1 | İbiş et al. (2015) |
| Golden Jackal 5 |  | AY289996 |  | Aggarwal et al. (2007) |
|  |  |  | AY289997 | Aggarwal et al. (2007) |

**Table S3.** Overview of the cytochrome *b* sequences used in the phylogenetic analysis. Two unique Himalayan wolf cytochrome *b* haplotypes (i.e. Himalayan wolf Cytochrome B 1, Himalayan wolf Cytochrome B 2) and one unique domestic dog haplotype (i.e. Domestic dog Humla Nepal B 1) were found in the samples collected in the study area in Humla (Nepal). Marked with an asterisk*** are sequences that are unpublished and have been directly submitted to NCBI GenBank by the respective authors. Reference sequences from the following publications were included: (3–13).

| **Haplotype** | **Unique Haplotypes Accession No.** | **Identical sequences Accession No.** | **Publication** |
| --- | --- | --- | --- |
| Himalayan wolf Cytochrome B 1 | KY996533 |  | This study |
| Himalayan wolf Cytochrome B 2 | KY996534 |  | This study |
|  |  | AY291431 | Aggarwal et al. (2007) |
| Domestic dog Humla Nepal B 1 | KY996532 |  | This study |
|  |  | KT447685 | Koepfli et al. (2015) |
|  |  | DQ480497 | Björnerfeldt et al. (2006) |
|  |  | DQ480491 | Björnerfeldt et al. (2006) |
|  |  | DQ480498 | Björnerfeldt et al. (2006) |
|  |  | DQ480495 | Björnerfeldt et al. (2006) |
|  |  | DQ480496 | Björnerfeldt et al. (2006) |
| Indian grey wolf 1 | AY291432 |  | Aggarwal et al. (2007) |
| Domestic dog 1 | KT447684 |  | Koepfli et al. (2015) |
|  |  | DQ480490 | Björnerfeldt et al. (2006) |
|  |  | DQ480494 | Björnerfeldt et al. (2006) |
|  |  | DQ480500 | Björnerfeldt et al. (2006) |
| Grey wolf Israel 1 | KT447705 |  | Koepfli et al. (2015) |
|  |  | KT447706 | Koepfli et al. (2015) |
|  |  | KT447700 | Koepfli et al. (2015) |
|  |  | KT447702 | Koepfli et al. (2015) |
|  |  | KT447709 | Koepfli et al. (2015) |
|  |  | KT447710 | Koepfli et al. (2015) |
|  |  | KT447707 | Koepfli et al. (2015) |
| Grey wolf Ukraine | KT447701 |  | Koepfli et al. (2015) |
| Grey wolf Israel 2 | KT447708 |  | Koepfli et al. (2015) |
|  |  | DQ480504 | Björnerfeldt et al. (2006) |
|  |  | DQ480505 | Björnerfeldt et al. (2006) |
| Grey wolf Canada 1 | KT447699 |  | Koepfli et al. (2015) |
| Grey wolf Oman | KT447703 |  | Koepfli et al. (2015) |
| Grey wolf Saudi Arabia 1 | KT447704 |  | Koepfli et al. (2015) |
| Domestic dog 2 | DQ480499 |  | Björnerfeldt et al. (2006) |
| Grey wolf Saudi Arabia 2 | DQ480506 |  | Björnerfeldt et al. (2006) |
| Grey wolf Canada 2 | DQ480508 |  | Björnerfeldt et al. (2006) |
| Grey wolf Russia | DQ480503 |  | Björnerfeldt et al. (2006) |
| Grey wolf Saudi Arabia 3 | DQ480507 |  | Björnerfeldt et al. (2006) |
| Domestic dog 3 | DQ480492 |  | Björnerfeldt et al. (2006) |
|  |  | DQ480493 | Björnerfeldt et al. (2006) |
|  |  | DQ480502 | Björnerfeldt et al. (2006) |
|  |  | DQ480501 | Björnerfeldt et al. (2006) |
| Tibetean Fox Cytochrome B | identical with KT033906 |  | Werhahn et al. (2016) |
| Coyote 1 | KT447695 |  | Koepfli et al. (2015) |
| Coyote 2 | DQ480511 |  | Björnerfeldt et al. (2006) |
|  |  | DQ480509 | Björnerfeldt et al. (2006) |
|  |  | DQ480510 | Björnerfeldt et al. (2006) |
|  |  | KT447697 | Koepfli et al. (2015) |
|  |  | KT447698 | Koepfli et al. (2015) |
|  |  | KT447696 | Koepfli et al. (2015) |
| Ethopian wolf | KT447693 |  | Koepfli et al. (2015) |
|  |  | KT447694 | Koepfli et al. (2015) |
|  |  | KT447692 | Koepfli et al. (2015) |
|  |  | KT447691 | Koepfli et al. (2015) |
| Side-striped Jackal | KT447687 |  | Koepfli et al. (2015) |
| African Wild dog | KT447689 |  | Koepfli et al. (2015) |
| Black-backed jackal | KT447688 |  | Koepfli et al. (2015) |
| Sechuran fox | KT447686 |  | Koepfli et al. (2015) |
| Red fox | KY996535 |  | This study |
| Dhole | KT447690 |  | Koepfli et al. (2015) |
| Golden jackal 1 | AY291433 |  | Aggarwal et al. (2007) |
| Golden jackal 2 | KT447713 |  | Koepfli et al. (2015) |
|  |  | KT447729 | Koepfli et al. (2015) |
|  |  | KT447732 | Koepfli et al. (2015) |
|  |  | KT447715 | Koepfli et al. (2015) |
|  |  | KT447731 | Koepfli et al. (2015) |
|  |  | KT447725 | Koepfli et al. (2015) |
|  |  | KT447718 | Koepfli et al. (2015) |
|  |  | KT447724 | Koepfli et al. (2015) |
|  |  | KT447719 | Koepfli et al. (2015) |
|  |  | KT447730 | Koepfli et al. (2015) |
|  |  | KT447757 | Koepfli et al. (2015) |
|  |  | KT447758 | Koepfli et al. (2015) |
|  |  | KT447756 | Koepfli et al. (2015) |
|  |  | KT447755 | Koepfli et al. (2015) |
|  |  | KT447754 | Koepfli et al. (2015) |
|  |  | KT447752 | Koepfli et al. (2015) |
|  |  | KT447751 | Koepfli et al. (2015) |
|  |  | KT447750 | Koepfli et al. (2015) |
|  |  | KT447749 | Koepfli et al. (2015) |
|  |  | KT447753 | Koepfli et al. (2015) |
|  |  | KT447726 | Koepfli et al. (2015) |
|  |  | KT447728 | Koepfli et al. (2015) |
| Golden jackal 3 | KT447748 |  | Koepfli et al. (2015) |
| Golden jackal 4 | KT447712 |  | Koepfli et al. (2015) |
| Golden jackal 5 | KT447727 |  | Koepfli et al. (2015) |
| African wolf 1 | KT447762 |  | Koepfli et al. (2015) |
|  |  | KT447761 | Koepfli et al. (2015) |
| African wolf 2 | KT447720 |  | Koepfli et al. (2015) |
|  |  | KT447723 | Koepfli et al. (2015) |
|  |  | KT447714 | Koepfli et al. (2015) |
|  |  | KT447716 | Koepfli et al. (2015) |
|  |  | KT447721 | Koepfli et al. (2015) |
|  |  | KT447722 | Koepfli et al. (2015) |
| African wolf 3 | KT447759 |  | Koepfli et al. (2015) |
|  |  | KT447760 | Koepfli et al. (2015) |
| African wolf 4 | KT447733 |  | Koepfli et al. (2015) |
|  |  | KT447745 | Koepfli et al. (2015) |
|  |  | KT447746 | Koepfli et al. (2015) |
|  |  | KT447747 | Koepfli et al. (2015) |
|  |  |  | Koepfli et al. (2015) |
| African wolf 5 | KT447735 |  | Koepfli et al. (2015) |
|  |  | KT447736 | Koepfli et al. (2015) |
|  |  | KT447739 | Koepfli et al. (2015) |
|  |  | KT447740 | Koepfli et al. (2015) |
|  |  | KT447741 | Koepfli et al. (2015) |
|  |  | KT447742 | Koepfli et al. (2015) |
|  |  | KT447743 | Koepfli et al. (2015) |
|  |  | KT447744 | Koepfli et al. (2015) |
|  |  | KT447738 | Koepfli et al. (2015) |
|  |  | KT447734 | Koepfli et al. (2015) |
| African wolf 6 | KT447717 |  | Koepfli et al. (2015) |
| African wolf 7 | KT447737 |  | Koepfli et al. (2015) |

**Table S4.** Primer sequences with references as used in the analysis of the Zinc-finger X-chromosomal (ZFX) and Y-chromosomal (ZFY) protein gene sequences.

| Oligo Name | Sequence (5' to 3') | Reference |
| --- | --- | --- |
| Zinc-Finger X-chromosomal (ZFX) and Y-chromosomal (ZFY) | | |
| U-ZF-2F | GACCTGAWTCCARRCAGTAC | (2) |
| U-ZF-2R | SCCACAAAWCATGCAAGG |  |
| C-ZFX-F | GTTCCCTTAAGGCAGGCATA | (1) |
| C-ZFX-R | AAAGCTTAAATCCACCTATGGAAA |  |
| C-ZFY-F | CAAGTTAGCATAAATTTGGTTTG |  |
| C-ZFY-R | TGTCTCTGCCTCTCTGTGTCTC |  |
| Canidpost-ZFYR | AAATTTCTTCACTCAGATGAAATAACA | (3) |

**
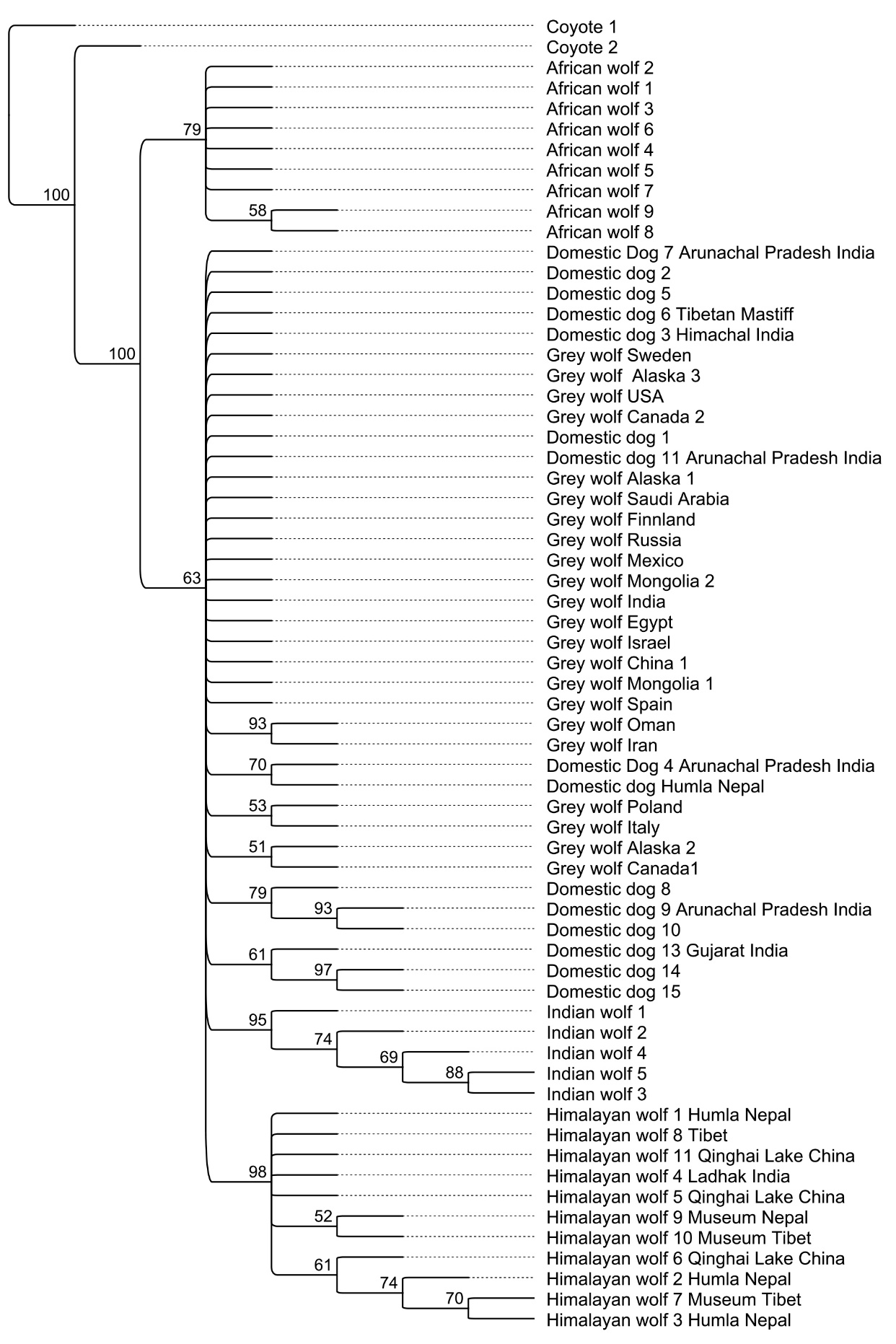
**

**Figure S1.** Phylogeny of 242bp D-loop mtDNA based on Neighbourhood joining with consensus support given in the branch nodes. The Himalayan wolf (green) is a monophyletic clade within the grey wolf-dog clade (blue) which includes also domestic dogs.


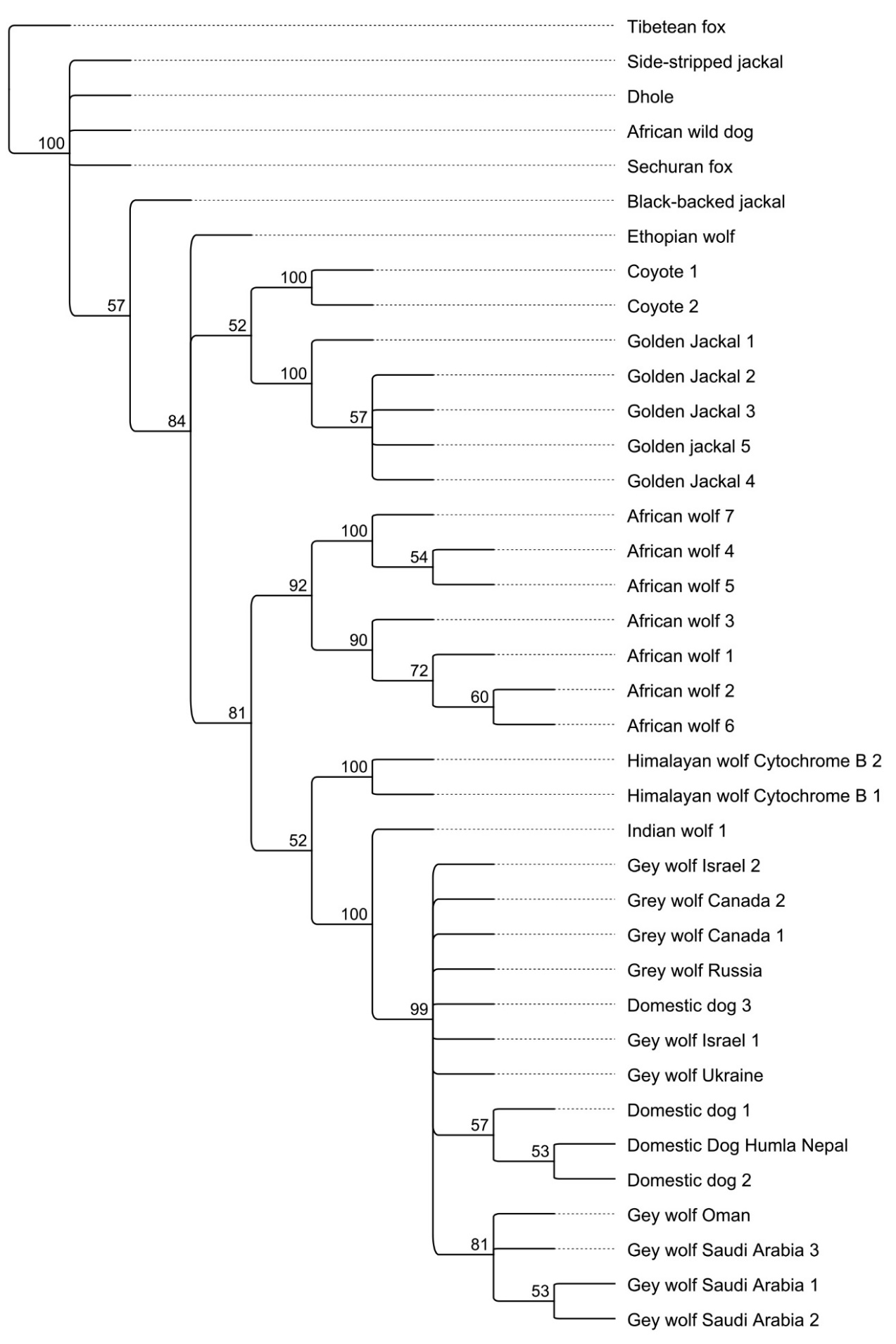


**Figure S2.** Phylogeny of 508bp cytochrome *b* mtDNA based on Neighbourhood joining with consensus support given in the branch nodes. The Himalayan wolf (green) is a monophyletic clade basal to the grey wolf-dog clade (blue) which includes also domestic dogs.

**REFERENCES**

1. Tsubouchi A, Fukui D, Ueda M, Tada K, Toyoshima S, Takami K, et al. Comparative molecular phylogeny and evolution of sex chromosome DNA sequences in the family Canidae (Mammalia: Carnivora). 2012;29(3):151–61.

2. Nakagome S, Pecon-Slattery J, Masuda R. Unequal Rates of Y Chromosome Gene Divergence during Speciation of the Family Ursidae. 2008;25(7):1344–56.

3. Koepfli K-P, Pollinger J, Godinho R, Robinson J, Lea A, Hendricks S, et al. Genome-wide Evidence Reveals that African and Eurasian Golden Jackals Are Distinct Species. Curr Biol. 2015 Aug 17;25(16):2158–65.

4. Aggarwal RK, Kivisild T, Ramadevi J, Singh L. Mitochondrial DNA coding region sequences support the phylogenetic distinction of two Indian wolf species. J Zool Syst Evol Res. 2007;45(2):163–172.

5. Björnerfeldt S, Webster MT, Vilà C. Relaxation of selective constraint on dog mitochondrial DNA following domestication. Genome Res. 2006 Jan 8;16(8):990–4.

6. Gaubert P, Bloch C, Benyacoub S, Abdelhamid A, Pagani P, Djagoun CAMS, et al. Reviving the African Wolf Canis lupus lupaster in North and West Africa: A Mitochondrial Lineage Ranging More than 6,000 km Wide. PLoS ONE. 2012 Aug 10;7(8):e42740.

7. Gottelli D, Marino J, Sillero-Zubiri C, Funk SM. The effect of the last glacial age on speciation and population genetic structure of the endangered Ethiopian wolf (Canis simensis). Mol Ecol. 2004 Aug 1;13(8):2275–86.

8. İbiş O, Aksöyek E, Özcan S, Tez C. A preliminary phylogenetic analysis of golden jackals (Canis aureus)(Canidae: Carnivora: Mammalia) from Turkey based on mitochondrial D-loop sequences. Vertebr Zool. 2015;391–7.

9. Ishiguro N, Inoshima Y, Shigehara N. Mitochondrial DNA Analysis of the Japanese Wolf (Canis Lupus Hodophilax Temminck, 1839) and Comparison with Representative Wolf and Domestic Dog Haplotypes. Zoolog Sci. 2009 Oct 30;26(11):765–70.

10. Rueness EK, Asmyhr MG, Sillero-Zubiri C, Macdonald DW, Bekele A, Atickem A, et al. The Cryptic African Wolf: Canis aureus lupaster Is Not a Golden Jackal and Is Not Endemic to Egypt. PLoS ONE. 2011 Jan 26;6(1):e16385.

11. Sharma DK, Maldonado JE, Jhala YV, Fleischer RC. Ancient wolf lineages in India. Proc R Soc Lond B Biol Sci. 2004;271(Suppl 3):S1–S4.

12. Thalmann O, Shapiro B, Cui P, Schuenemann VJ, Sawyer SK, Greenfield DL, et al. Complete Mitochondrial Genomes of Ancient Canids Suggest a European Origin of Domestic Dogs. Science. 2013 Nov 15;342(6160):871–4.

13. Waters S, El Harrad A, Amhouch Z, Taiqui L, Senn H. Distribution update DNA analysis confirms African wolf in Morocco. Canid Biol Conserv. 2015;18(5).
